# Anorectic and aversive effects of GLP-1 receptor agonism are mediated by brainstem cholecystokinin neurons, and modulated by GIP receptor activation

**DOI:** 10.1016/j.molmet.2021.101407

**Published:** 2021-11-26

**Authors:** Alessia Costa, Minrong Ai, Nicolas Nunn, Isabella Culotta, Jenna Hunter, Mehdi Boutagouga Boudjadja, Lourdes Valencia-Torres, Gabriella Aviello, David J. Hodson, Brandy M. Snider, Tamer Coskun, Paul J. Emmerson, Simon M. Luckman, Giuseppe D'Agostino

**Affiliations:** 1 Faculty of Biology, Medicine and Health, University of Manchester, Manchester, UK; 2 Lilly Research Laboratories, Eli Lilly & Company, Indianapolis, IN, United States; 3 The Rowett Institute, University of Aberdeen, Aberdeen, UK; 4 Department of Pharmacy, University of Naples Federico II, Naples, Italy; 5 Institute of Metabolism and Systems Research University of Birmingham &Centre for Endocrinology, Diabetes and Metabolism, Birmingham Health Partners, Birmingham, UK

**Keywords:** Brain, Appetite, Nausea, Glucagon-like peptide-1, Glucose-dependent insulinotropic polypeptide, Cholecystokinin, Area postrema, Nucleus of the solitary tract

## Abstract

**Objective:**

Glucagon-like peptide-1 receptor agonists (GLP-1RAs) are effective medications to reduce appetite and body weight. These actions are centrally mediated; however, the neuronal substrates involved are poorly understood.

**Methods:**

We employed a combination of neuroanatomical, genetic, and behavioral approaches in the mouse to investigate the involvement of caudal brainstem cholecystokinin-expressing neurons in the effect of the GLP-1RA exendin-4. We further confirmed key neuroanatomical findings in the non-human primate brain.

**Results:**

We found that cholecystokinin-expressing neurons in the caudal brainstem are required for the anorectic and body weight-lowering effects of GLP-1RAs and for the induction of GLP-1RA-induced conditioned taste avoidance. We further show that, while cholecystokinin-expressing neurons are not a direct target for glucose-dependent insulinotropic peptide (GIP), GIP receptor activation results in a reduced recruitment of these GLP-1RA-responsive neurons and a selective reduction of conditioned taste avoidance.

**Conclusions:**

In addition to disclosing a neuronal population required for the full appetite- and body weight-lowering effect of GLP-1RAs, our data also provide a novel framework for understanding and ameliorating GLP-1RA-induced nausea — a major factor for withdrawal from treatment.

## Highlights

• CCK^AP/NTS^ neurons are required for the full anorectic and body weight-lowering effect of GLP-1 receptor agonists.
• GLP-1 receptor agonists promote the formation of conditioned taste avoidance by activating CCK^AP/NTS^ neurons.
• CCK^AP/NTS^ neurons are not activated in response to GIP receptor agonists.
• GIP receptor agonists reduce GLP-1 receptor agonist-induced neuronal responses in the caudal brainstem.
• GIP receptor agonists reduce GLP-1 receptor agonist-induced conditioned taste avoidance.

## 1 Introduction

Glucagon-like peptide-1 (GLP-1) and glucose-dependent insulinotropic polypeptide (GIP) are known as incretins, which are released from the gut into the bloodstream postprandially and enhance glucose-dependent insulin secretion via activation of the GLP-1 receptor (GLP-1R) and the GIP receptor (GIPR), respectively [1]. Several GLP-1R agonists (GLP-1RA) with improved pharmacokinetic properties have been developed and are currently in clinical use to treat type 2 diabetes and obesity [1,2]. In addition to improving glucose metabolism, GLP-1RAs potently suppress appetite and body weight. These anorectic and body weight-lowering effects are thought to be mediated by central mechanisms [[3], [4], [5], [6], [7], [8]], as indicated also by human studies [[9], [10], [11], [12]]. However, the neuronal substrates that mediate these effects are still poorly understood.

In addition to understanding the appetite-suppressing effects of GLP-1RAs, identifying these neuronal substrates will also help in understanding the known side effects commonly associated with GLP-1R agonists, which represent a barrier to the full therapeutic exploitation of these medications. For example, in clinical trials with liraglutide or semaglutide for obesity, patient reports of nausea (40.2% and 44.2%, respectively) and vomiting (16% and 24.8%, respectively) were significantly higher than those reported in the respective placebo groups [13,14].

GLP-1R is distributed across several brain regions [12,[15], [16], [17]] and among these, the brainstem (which includes the dorsal vagal complex and the parabrachial nucleus) has long been considered an important site of action for GLP-1RAs [4,6,[18], [19], [20]]. The caudal brainstem has been shown to be sufficient for mediating anorexia elicited by GLP-1RAs in decerebrated rats [6]. Furthermore, site-specific injections of GLP-1RAs in the dorsal vagal complex suppress intake and motivational aspects of feeding in rats [18], while inhibition of the parabrachial nucleus — a target for dorsal vagal complex neurons — significantly attenuates GLP-1RA-induced anorexia in mice and rats [21,22].

As this and other evidence point to the caudal brainstem as an important central hub mediating the anorectic effects of GLP-1R activation [4,6,[18], [19], [20]], here we identify the neurons involved. We and others have previously described a population of dorsal vagal complex neurons defined by the expression of cholecystokinin (CCK) [[23], [24], [25]]. CCK-expressing neurons are present in the nucleus of the solitary tract (NTS) and area postrema (AP; hereafter referred to as CCK^AP/NTS^). CCK^AP/NTS^ neuron activation occurs postprandially and potently suppresses appetite [[23], [24], [25]]. Using complementary experimental approaches, we found that CCK^AP/NTS^ are responsive to GLP-1RAs and represent a critical mediator of their anorectic effects, as well as their ability to induce nausea (measured as conditioned taste avoidance; CTA). We further show that this population of GLP-1RA-responsive neurons is not a direct target for GIP. However, co-administration of GLP-1 and GIP analogues results in a reduced recruitment of these brainstem neurons and a selective reduction of CTA.

## 2 Material and methods

### 2.1 Animals

*CCK-ires-Cre (STOCK Ccktm1.1(cre)Zjh/J)*and R26-loxSTOPlox-eYFP (Ai3; B6.Cg-Gt (ROSA)26Sor^tm3(CAG−EYFP)Hze^/J) were obtained from Jackson Laboratories and fed standard mouse chow and water *ad libitum*, unless otherwise noted. C57BL/6JRj mice (7–8 weeks of age) were obtained from Janvier Laboratories (France) and maintained on standard mouse chow. On a C57BL/6J genetic background, *Glp1r−/−* and *Gipr−/−* mice [26,27] were obtained from Taconic (Petersburgh, NY, USA). These mice were housed at 22–24 °C with a 12-h light/12-h dark cycle. All experimental procedures were performed in accordance with the UK Animals (Scientific Procedures) Act 1986 and local ethical review, or in compliance with Eli Lilly and Company's Institutional Animal Care and Use Committee.

### 2.2 Drugs and viral vectors

All drugs were purchased from Tocris Bioscience (Bristol, UK). Devazepide (Tocris Bioscience, Cat. No. 2304) was dissolved in Dimethylsulfoxide (DMSO), further diluted with sterile saline and administered at 1 mg kg-1, IP, 40 min before Exendin-4. All other drugs for in vivo use were prepared in sterile saline and administered intraperitoneally (IP).

Fluoro-Gold (hydroxystilbamidine, 4% w/v solution in water; Invitrogen, Thermo Fisher, MA) was injected into mice anaesthetized with isoflurane (2–3% in oxygen) and placed in a stereotaxic frame. The skull was exposed and holes were drilled at the site of injection. Fluoro-Gold was delivered unilaterally via a glass micropipette affixed to a Nanoject II Auto Nanoliter Injector (Drummond Scientific Company, PA) using co-ordinates as determined in the Mouse Brain Atlas: (Paxinos and Franklin, 2004) elPBN, −4.9 mm A/P, −1.7 mm M/L, −3.8 mm D/V from bregma (150 nl); PVH, - 0.7 mm A/P; −0.3 mm M/L; −5.5 mm D/V (50 nl). All animals were left to recover for 2 weeks to allow axonal transport before being transcardially perfused (see below).

Viral injections into the AP/NTS were performed as described previously with minor modifications [23]. Briefly, mice were anaesthetized with a mixture of ketamine and xylazine dissolved in saline (80 and 10 mg/kg, respectively). Mice were placed in a stereotaxic frame, an incision was made at the level of the cisterna magna, and neck muscles were carefully retracted. Following dura incision, the obex served as a reference point for injections with a glass micropipette. AP/NTS coordinates were approximately 0.2 mm A/P, 0 and ± 0.2 mm M/L, −0.2 mm D/V from obex. About 100 nl of virus were delivered during each of the three microinjections. The animals were administered analgesia (5 mg/kg Carprofen, s.c.) for 2 days post-operatively and they were given a minimum of 14 days to recover before night-time feeding measurements. AAVs were obtained from Addgene (Watertown, MA). AAVs expressing TeTLC-eGFP were obtained from the Vector Core of the Stanford University (Stanford, CA).

### 2.3 Electrophysiology

*Cck*^Cre^::*eYFP* mice were euthanized by decapitation, and the brain was removed and placed in ice-cold oxygenated aCSF containing (in mM): 95 NaCl, 1.8 KCl, 1.2 KH2PO4, 7 MgSO4, 26 NaHCO3, 0.5 CaCl2, 15 glucose, and 50 sucrose (osmolality 300–310 mOsm). About 250-μm-thick slices containing the dorsal vagal complex were cut on a vibratome (Campden Instruments, Loughborough, UK), and kept in the cutting solution for >1 h before being placed in the recording solution. For recordings, slices were perfused at room temperature with oxygenated aCSF containing the following concentrations (in mM): 127 NaCl, 1.8 KCl, 1.2 KH2PO4, 1.3 MgSO4, 26 NaHCO3, 2.4 CaCl2, and 5 glucose. Fluorescent neurons were visualized with an Olympus BX51 microscope with DIC optics and a GFP fluorescence filter. Neurons were patched using 7–8 MΩ pipettes containing (in mM): 130 K-gluconate, 10 KCl, 2 MgCl2, 10 HEPES, 0.5 EGTA, 2 K2ATP, and NaGTP. Current clamp data were acquired with a CED 1401 interface (CED, Cambridge, UK) and sampled at 30 kHz using an Axoclamp 2A bridge mode amplifier (Molecular Devices, San Jose, CA). Exendin-4 (Tocris, Bristonl, UK) was diluted in the recording solution to 1 μM and bathed on a slice at 1–2 ml/min using a gravity-driven system.

### 2.4 Energy balance and body weight studies

*Dark-cycle food intake:* Mice were injected with drugs 30 min prior to the onset of the dark cycle. At the onset of darkness, food was returned. Intake was measured either manually or automatically using either the TSE Phenomaster system (TSE, Germany) or the Promethion Core System (Sable System International, Germany). *Post-fast re-feed*: Mice were fasted overnight. The following morning, mice were IP treated with drugs, food was returned 30 min later and intake was recorded manually. *Daily treatment*: Mice were IP treated twice a day, near the onset of the dark and light cycles. Body weight values were recorded daily prior to the dark cycle. Indirect calorimetry and locomotor activity measurements were taken using the TSE Phenomaster system (TSE, Germany).

### 2.5 Optogenetic and behavioral assays

Fiber optic cables (1.5 m long, 200 μm diameter, 0.22 NA; Doric Lenses, Franquet, Quebec, Canada) were firmly attached to the implanted fiber optic cannulae with zirconia sleeves (Doric Lenses). Photostimulation was programmed using a pulse generator software (Prizmatix, Givat-Shmuel, Israel) that controlled a blue light laser (473 nm; Laserglow, Toronto, Canada) via a USB-TTL interface (Prizmatix). Photostimulation for feeding experiments: light pulse trains with 10-ms pulse width, 30 Hz, 1 s on, 0.5 s off. Light power was adjusted such that the light power exiting the fiber optic cable was at least 10 mW using a digital optical power meter (PM100D, Thorlabs) and an online light transmission calculator for brain tissue (http://web.stanford.edu/group/dlab/cgi-bin/graph/chart.php).

*Real-time place preference:* Mice were tested for real-time place preference in a model in which one chamber was paired with 30-Hz photostimulation and the other, identical chamber, resulted in no photostimulation. The total test duration was 20 min. Time spent in the photostimulation versus non-photostimulation zones was recorded via a CCD camera interfaced with Any-maze software (Stoelting, Wood Dale, IL).

*Conditioned taste avoidance (CTA):* On day 1 of the study, animals were food-deprived overnight for the optogenetic stimulation experiment. The following morning (day 2), the animals were given flavored food pellets for 30 min and their intake was measured. At the end of the 30 min, animals received optogenetic stimulation for additional 30 min. On day 3, mice were again food-deprived overnight before being given the same flavored food pellets used for conditioning on day 4. CTA was also assessed using a one-bottle CTA protocol in sated mice. The mice were given either of two flavored milkshake solutions (banana or chocolate) daily for 2 h. Over 3–5 days, mice spontaneously displayed stable consumption. For the conditioning, on day 8, the flavor that mice had consumed during the training session was swapped with the other, novel one (conditioned stimulus; CS+) for 4 h. The consumption of the novel flavor was paired with a systemic injection of EX-4 (US). Training and novel flavors were counterbalanced. Two days following the conditioning session, mice were given the CS+ flavor again and their intake measured for 2 h.

### 2.6 Histology

For immunofluorescent staining, mice were transcardially perfused with phosphate buffered saline (PBS), followed by 10% neutral buffered formalin (SigmaAldrich). Brains were extracted, post-fixed in 10% neutral buffered formalin at 4C, cryoprotected in 20% sucrose at 4C, and then sectioned coronally on a freezing sliding microtome at 25 μm. Tissue was processed using standard protocols, as previously described [23]. Briefly, sections were washed in PBS before being blocked in 0.5% BSA/0.25% Triton X-100 in PBS for 1 h at room temperature. Tissue was incubated overnight at room temperature in blocking buffer containing the primary antibodies: rabbit anti-FOS (Abcam, Cambridge, UK; Cat. No. ab190289; diluted 1/1000), chicken anti-GFP (Abcam, Cambridge, UK; Cat. No. ab13970; diluted 1/1000), and goat anti-mCherry (Sicgen, Cat. No. ab0040-200; diluted 1/5000). The next day, sections were washed in PBS then incubated in blocking buffer containing secondary antibodies: donkey anti-rabbit (Abcam, Cambridge, UK; Cat. No. ab150076Alexa Fluor 594; diluted 1/1000); Goat anti-chicken (Abcam, Cambridge, UK; Cat. No. ab150169Alexa Fluor 488; diluted 1/1000); and Donkey anti-goat (Abcam, Cambridge, UK; Cat. No. ab150132Alexa Fluor 594; diluted 1/1000) for 2 h. Sections were then washed in PBS and mounted on slides, cover slipped and visualized on a Zeiss Axiomanager. D2 upright microscope (Zeiss, Oberkochen, Germany) and images were captured with a Coolsnap HQ1 camera (Photometics, AZ) using Micromanager software v1.4.23 (https://imagej.net/Micro-Manager). Specific band pass filter sets for DAPI, FITC, and Texas Red were used to prevent bleed through from one channel to the next. All images were processed and analysed using Fiji ImageJ (https://fiji.sc/). For FOS quantification, 3–4 sections per mouse at the level of the AP were selected, and cells were counted manually in the AP and the adjacent NTS.

For fluorescent *in situ* hybridization, mRNA was detected using RNAscope Multiplex Fluorescent Assay reagent kits (Advanced Cell Diagnostics, Inc, Newark, CA), according to the manufacturer's instructions, at Gubra (Hørsholm, Denmark). Slides were counter-stained with DAPI to identify cellular nuclei. Slides were scanned under a 20X objective on an Olympus VS120 Fluorescent scanner. Mice were terminally anaesthetized and their brains were removed and snap frozen on crushed dry ice. Around four or five 10-μm-thick tissue sections at the level of the AP were collected. For quantification, three sections per mouse at the level of the AP were selected, and cells were counted manually in the AP and the adjacent NTS. Tissue blocks of an adult (6–7 years of age) cynomolgus monkey brain containing the area postrema were obtained from MediTox (Czech Republic). A block containing the brainstem and cerebellum was fixated in 10% neutral buffered formalin for 24 h at room temperature, followed by 24 h at 4 °C before being transferred to 70% ethanol. Paraffin-infiltrated and embedded tissue blocks covering the area postrema were cut on a microtome into a series of 5-μm-thick sections and collected. For quantification, 2–3 sections at the level of the AP were selected and cells were counted manually in the AP and the adjacent NTS.

### 2.7 Statistics

Statistical analyses were performed using Prism 6 (Graphpad Software, Inc). Data were analyzed using t-test, one-way ANOVA, or two-way ANOVA with *post hoc* comparisons, wherever appropriate. The data are presented as mean +/− SEM, with statistical significance set at p < 0.05.

## 3 Results and discussion

### 3.1 CCK^AP/NTS^ neurons are responsive to GLP-1R agonists

**Figure 1:**
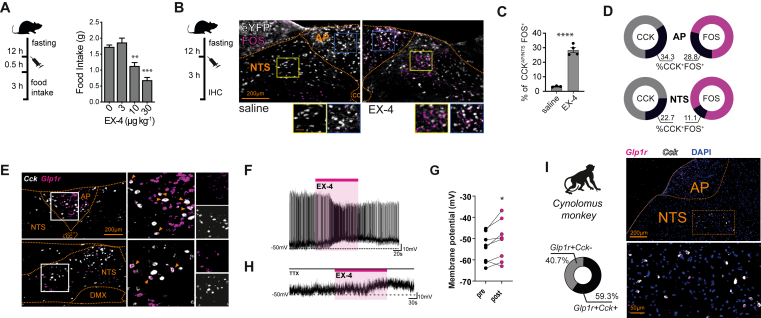
**CCK**^**AP/NTS**^**neurons are responsive to GLP-1R agonist Exendin-4. (A)**Exendin-4 (EX-4) suppresses food intake (n = 8–9; Treatment: F_(3, 31)_ = 21.81, p < 0.0001. Post hoc ∗p = 0.0006, ∗∗∗p < 0.0001), **(B)** Representative images, and **(C)** quantification of FOS expression in CCK^AP/NTS^ neurons, identified as eYFP-expressing cells in *Cck*^Cre^::eYFP mice, following saline or EX-4 administration (n = 3–4; t (5) = 11.57, p < 0.0001). (**D**) Distribution of activated CCK^AP/NTS^ neurons between AP and NTS and in relation to total FOS-expressing cells. **(E)**Representative fluorescence *in situ* hybridization (FISH) labeling of endogenous *Cck* and *Glp1r* mRNAs in the AP and the NTS of the mouse (n = 3), **(F)** Representative trace, and **(G)** quantification of changes in membrane potential recorded upon bath application of EX-4 (1 μM; *t* (7) = 2.692, p = 0.0310). **(H)** Representative trace recorded upon application of EX-4 in the presence of tetrodotoxin (TTX; 1 μM). **(I)** Representative FISH labeling of*Cck* and *Glp1r* mRNAs in the NTS of the of the *Cynomolgus* monkey (n = 2). Data are presented as mean ± SEM. AP, area postrema; NTS, nucleus of the solitary tract; cc, central canal; DMX, dorsal motor nucleus of the vagus; and IHC, immunohistochemistry.

As reported previously, CCK-expressing cells are present within the NTS and the AP [[23], [24], [25], [26], [27]]. To determine whether CCK^AP/NTS^ cells are responsive to systemically administered GLP-1R agonists, we treated *Cck*^Cre^::eYFP mice with the selective and stable GLP-1R agonist exendin-4 (EX-4) and assessed FOS expression — a surrogate marker of neuronal activation. In CCK^AP/NTS^ cells, an injection of EX-4 at a dose sufficient to reduce food consumption (10 μg kg^−1^, IP; Figure 1A) elicits a significant increase in FOS expression when compared to a control vehicle injection (Figure 1B,C). About 34% of CCK-eYFP cells in the AP express FOS (EX-4 34.2% ± 3.0 *vs* saline 4.1% ± 0.2), accounting for 29 ± 4% of total FOS-expressing cells (Figure 1D). About 23% of CCK-eYFP cells located in the adjacent NTS also express FOS in response to EX-4 (EX-4 22.7% ± 2.0 *vs* saline 2.6% ± 0.5), accounting for 11 ± 1% of total FOS-expressing cells (Figure 1D). Thus, a significant portion of CCK^AP/NTS^ neurons express the neuronal activity marker FOS in response to the systemic administration of the GLP-1R agonist EX-4.

Since the AP and the NTS are readily accessible to GLP-1R ligands when administered systemically [4,19,28,29], we asked whether CCK^AP/NTS^ neurons express GLP-1R. We performed dual-label fluorescence *in situ* hybridization histology (FISH) in wild-type mice and found co-expression of *Cck* and *Glp1r* mRNAs within both the AP and the NTS (Figure 1E). The majority of *Cck* mRNA-expressing cells in the AP express *Glp1r* mRNA (69.2% ± 5.4), accounting for about 25% of the *Glp1r* mRNA expressing cells in that structure (24.1% ± 2.0). Although we observed co-localization, the *Glp1r* mRNA signal in the NTS was weaker, making it difficult to accurately quantify the degree of co-localization between *Cck* and *Glp1r* mRNAs. However, using whole-cell electrophysiological recordings of fluorescently identified neurons, we found that EX-4 increases the rate of action potential firing in CCK neurons with basal firing activity (Figure 1F) and significantly increases depolarization overall (Figure 1G). EX-4-induced depolarization endures in the presence of the synaptic blocker tetrodotoxin (TTX) in 5 out of 7 CCK neurons recorded in the NTS (Figure 1H), indicating that EX-4 can directly activate CCK^NTS^ neurons via a post-synaptic mechanism. Importantly, we found co-expression of *Cck* and *Glp1r* mRNAs within the dorsal vagal complex of the Cynomolgus monkey (Figure 1I). More than half (59.3% ± 8.6) of the *Glp1r* mRNA expressing cells in the NTS also express *Cck* mRNAs (namely, 67.9% and 50.7% from two subjects analyzed). We observed, however, lower expression of *Cck* mRNA in the AP, suggesting some phylogenetic differences between mice and non-human primates with regards to CCK neuron distribution within the dorsal vagal complex.

Together, these data suggest that a subpopulation of CCK^AP/NTS^ neurons express GLP-1R and could, therefore, be a direct target of GLP-1RAs. However, bearing in mind the caveats associated with the histology methods employed, it is possible that CCK^AP/NTS^ neurons could also respond to GLP-1R agonists via synaptic inputs. These inputs might include vagal afferent neurons [7,30], AP neurons synapsing onto neighboring NTS neurons [31], as well as inputs from other central GLP-1RA-responsive cells.

### 3.2 CCK^AP/NTS^ neurons are required for the anorectic and bodyweight lowering effect of GLP-1R agonists

**Figure 2:**
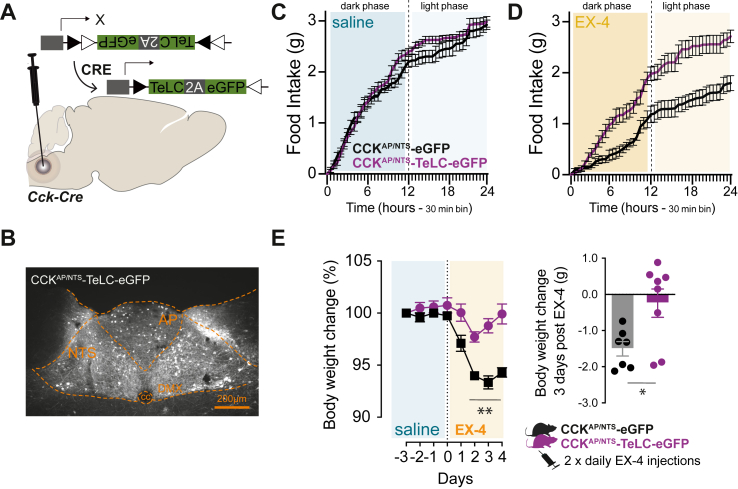
**CCK**^**AP/NTS**^**neurons are required for the anorectic and body-weight lowering effects of GLP-1R agonists.** (**A**) Schematic of the strategy to inhibit CCK^AP/NTS^ neurons using Cre-dependent AAV expressing an eGFP-fused tetanus-toxin-light-chain (TeLC) and (**B**) TeLC-eGFP expression in CCK^AP/NTS^ neurons.**(C)** Food intake in control (CCK^AP/NTS^-eGFP; n = 7) and CCK^AP/NTS^-TeLC-eGFP (n = 8) mice injected with saline (F_(1,13)_ = 1.55, p = 0.2350) and **(D)** EX-4 (10 μg kg^−1^, IP; F_(1,13)_ = 19.02, p = 0.0008).(**E**) EX-4 (20 μg kg^−1^, IP, twice daily) suppresses body weight in control CCK^AP/NTS^-eGFP (n = 7), but not in CCK^AP/NTS^-TeLC-eGFP (n = 8) mice (Treatment: F_(1,13)_ = 56.23, p < 0.0001; Time: F_(7,91)_ = 22.21, p < 0.0001; Interaction: F_(7,91)_ = 7.48, p < 0.0001; two-way ANOVA. Delta BW: t (13) = 2.745,p = 0.0167). Data are presented as mean ± SEM. See also, Supplemental Fig. 1.

Notwithstanding the mechanism, our data suggest that CCK^AP/NTS^ neurons are activated in response to the GLP-1RA EX-4. We and others have shown previously that activation of CCK^AP/NTS^ neurons occurs postprandially and that their activation suppresses appetite [[23], [24], [25]]. Therefore, we tested whether CCK^AP/NTS^ neurons have a role in the anorectic action of GLP-1RAs. To do this, we employed a loss-of-function approach and targeted CCK^AP/NTS^ neurons with a Cre-dependent AAV expressing an eGFP-fused tetanus-toxin-light-chain (TeLC) in *Cck*^Cre^ mice (CCK^AP/NTS^-TeLC; Figure 2A,B), as previously performed [24]. TeLC is a protease selective for the synaptic protein synaptobrevin-2, whose expression in neurons results in the inhibition of synaptic transmission. As expected, EX-4 (10 μg kg^−1^, IP) suppresses food intake when administered to control mice targeted with an AAV expressing a Cre-dependent eGFP (CCK^AP/NTS^-eGFP; Figure 2C–E). However, we found that the same treatment failed to suppress food intake in CCK^AP/NTS^-TeLC mice (Figure 2C–E). These results were replicated in three independent cohorts of mice and held true even when a three-times higher dose of EX-4 was used (30 μg kg^−1^, IP; data not shown).

We also tested the effect of sub-chronic EX-4 administration on body weight in a cohort of CCK^AP/NTS^-TeLC and CCK^AP/NTS^-eGFP mice. In CCK^AP/NTS^-eGFP mice, twice-daily injection of EX-4 (20 μg kg^−1^, IP) is sufficient to decrease body weight, achieving a peak of about 5% weight loss within 3 days of treatment. This effect was absent in CCK^AP/NTS^-TeLC mice (Figure 2F). Thus, CCK^AP/NTS^ neurons also mediate the effect of EX-4 on body weight when this drug is administered sub-chronically.

In the steady state, we found no significant phenotypic differences between CCK^AP/NTS^-TeLC and CCK^AP/NTS^-eGFP mice. Overall, there was no difference in feeding, energy expenditure, locomotor activity, and body weight (Supplemental Fig. 1). These data are in line with recent findings using similar loss-of-function approaches to inhibit CCK^AP/NTS^ neurons [24]. We observed a small, but significant and reproducible increase in water intake in CCK^AP/NTS^-TeLC mice (Supplemental Fig. 1), which complements recent data suggesting that activation of CCK^NTS^ neurons also suppresses water intake [32].

Thus, while CCK^AP/NTS^ neurons seem dispensable for maintaining energy balance under normal conditions, our data reveal that these cells are required for the full anorectic and body weight-lowering effects of the GLP-1RA EX-4.

### 3.3 GLP-1R agonists elicit nausea and behavioral avoidance via CCK^AP/NTS^ neurons

**Figure 3:**
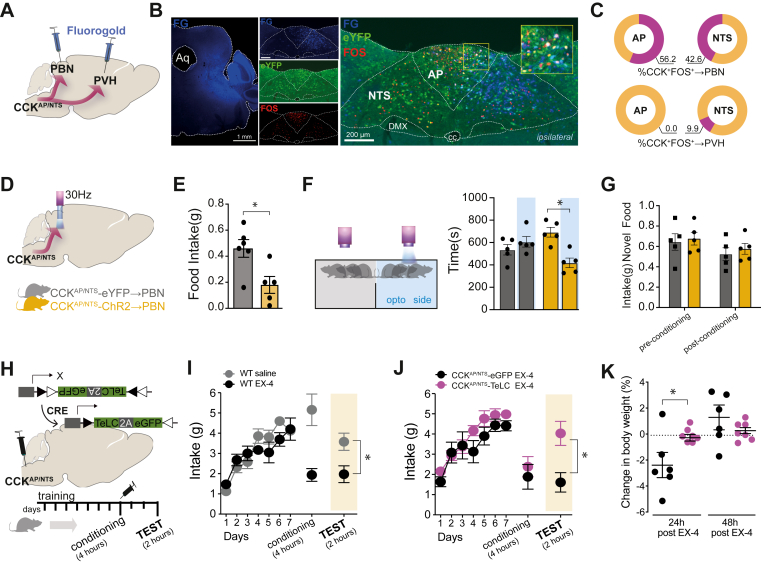
**Exendin-4 induces behavioral avoidance via CCK**^**AP/NTS**^**neurons.** (**A**) Schematic of the strategy to identify CCK^AP/NTS^ neurons projecting (→) to the parabrachial nucleus (PBN) or paraventricular nucleus of the hypothalamus (PVH) using Fluoro-Gold (FG) retrograde tracing. (**B**) Representative images showing CCK^AP/NTS^→PBN neurons activated by EX-4. (**C**) Quantification of CCK^AP/NTS^→PBN and CCK^AP/NTS^→PVH neurons activated by EX-4 (n = 3). (**D**) Schematic of the optogenetic strategy to stimulate CCK^AP/NTS^→PBN.**(E)**Stimulation of CCK^AP/NTS^→PBN reduces food intake in fasted mice (n = 5–6; t (9) = 2.936, p = 0.0166) and (**F**) induces place avoidance in a real-time place preference assay (t (8) = 4.485, p = 0.0020), but (**G**) it does not induce conditioned taste avoidance (CTA).(**H**) Schematic of the strategy to silence CCK^AP/NTS^ using TeLC. (**I**) EX-4 (30 μg kg^−1^, IP) induces CTA in wild-type mice (t (14) = 3.144, p = 0.0072). (**J**) EX-4 induces CTA in control CCK^AP/NTS^-eGFP (n = 6), but not in CCK^AP/NTS^-TeLC-eGFP (n = 7) mice (t (11) = 3.087, p = 0.0103). (**K**) EX-4 induces body weight loss in control CCK^AP/NTS^-eGFP (n = 6), but not in CCK^AP/NTS^-TeLC-eGFP (n = 7) mice (24 h: t (11) = 2.603, ∗p = 0.0246; 48 h:t (11) = 1.162, p = 0.270). Data are presented as mean ± SEM. See also, Supplemental Fig. 2.

Next, we began delineating the circuit through which CCK^AP/NTS^ neurons relay the EX-4 signal to other brain regions. We and others have previously shown that activation of CCK^NTS^ neurons reduces appetite through at least two parallel efferent projections, which encode appetitive and aversive valences and target the paraventricular nucleus of the hypothalamus (PVH) or the hindbrain parabrachial nucleus (PBN), respectively [23,25,33]. It is highly unlikely that any CCK^AP/NTS^ neurons will project to both of these areas: AP neurons do not project to the PVH [34], and NTS neurons projecting to either the PVH or PBN have very few, if any, collaterals [35,36]. Therefore, we asked to what extent administration of EX-4 recruits these two circuits. To do this, we repeated EX-4 treatment and FOS studies in *Cck*^Cre^::*eYFP* mice previously injected with the retrograde tracer, Fluoro-Gold, into the PVH or PBN (Figure 3A) to identify discrete CCK^AP/NTS^ neurons projecting to the PVH (CCK^AP/NTS^→PVH) or the PBN (CCK^AP/NTS^→PBN). We found that only about 10% of CCK^AP/NTS^ neurons (9.9% ± 3.7) expressed FOS following the EX-4 treatment project to the PVH. PVH-projecting CCK neurons were only observed in NTS (Supplemental Fig. 2). By contrast, we found that a large proportion of CCK^AP/NTS^ neurons (56,2% ± 13.9 in the AP and 42.6% ± 7.0 in the NTS) expressing FOS following EX-4 treatment projects to the PBN ipsilaterally (Figure 3B), indicating that systemic administration of EX-4 primarily recruits the CCK^AP/NTS^→PBN circuit (Figure 3C). This finding is also consistent with previous reports, underscoring the importance of PBN neurons to the anorectic effect of EX-4 [36,37].

The CCK^AP/NTS^→PBN circuit encodes negative valence [33]. To activate the CCK^AP/NTS^→PBN circuit and mimic the effect of EX-4 on this pathway, we targeted CCK^AP/NTS^ neurons with a Cre-dependent AAV expressing the excitatory channelrodopin-2 (ChR2) in *Cck*^Cre^ mice and implanted an optic fiber above the PBN (Figure 3D). Optogenetic activation of CCK^AP/NTS^→PBN terminals is sufficient to reduce food consumption (Figure 3E) and is aversive, as assessed using a real-time place preference assay (Figure 3F). Given that treatment with GLP-1RAs is associated with nausea in humans and can produce conditioned taste avoidance (CTA) in rodents [37], we reasoned that this CCK^AP/NTS^→PBN circuit could also be sufficient to induce CTA when the optogenetic stimulation is used as an unconditioned stimulus (US). However, we found that, immediately following the consumption of a novel food, optogenetic activation of the CCK^AP/NTS^→PBN does not promote CTA (Figure 3G), confirming data from similar experiments [33]. Thus, while the CCK^AP/NTS^→PBN circuit is a primary target of EX-4 and, when activated, is sufficient to suppress appetite and to encode negative valence, this circuit is not sufficient to induce CTA.

The absence of CTA following CCK^AP/NTS^→PBN stimulation raised the question as to whether CCK^AP/NTS^ neurons have a role in the formation of EX-4-induced CTA. To answer this question, we generated another cohort of CCK^AP/NTS^-TeLC and CCK^AP/NTS^-eGFP mice, as described above, and employed a simple, one-bottle CTA protocol in sated mice. Over 3–5 days, mice spontaneously displayed stable consumption of either of two flavored milkshake solutions. The flavor was swapped with the other, novel one (conditioned stimulus; CS) on day 8 and paired with a systemic injection of EX-4 (US; Figure 3H). We confirm that EX-4 (30 μg kg^−1^, IP) induces CTA in wild-type mice (Figure 3I). However, we found that while EX-4 also induced CTA in CCK^AP/NTS^-eGFP mice, this effect is almost completely absent in CCK^AP/NTS^-TeLC mice (Figure 3J). There was no difference in acute intake during the conditioning step of this protocol, possibly due to the involvement of other central mechanisms that control consumption of palatable diets that remain responsive in this experimental setting. Nonetheless, confirming the importance of CCK^AP/NTS^ neurons, EX-4 caused the expected body-weight loss in CCK^AP/NTS^-eGFP mice, recorded 24 h after the conditioning phase, but not in CCK^AP/NTS^-TeLC mice (Figure 3K).

Thus, CCK^AP/NTS^ neurons appear to be necessary for the formation of CTA following EX-4 treatment. Given that CCK^AP/NTS^→PBN activation does not seem to be sufficient to induce CTA, and that CCK^NTS^→PVH activation is not aversive [23,33], these findings suggest that an alternative (or additional) efferent projection pathway(s) from CCK^AP/NTS^ neurons is required for the formation of CTA following EX-4 treatment and warrant more circuit-specific studies in the future.

### 3.4 GIP analogues oppose the action of GLP-1R agonist to recruit CCK^AP/NTS^ and induce behavioral avoidance

**Figure 4:**
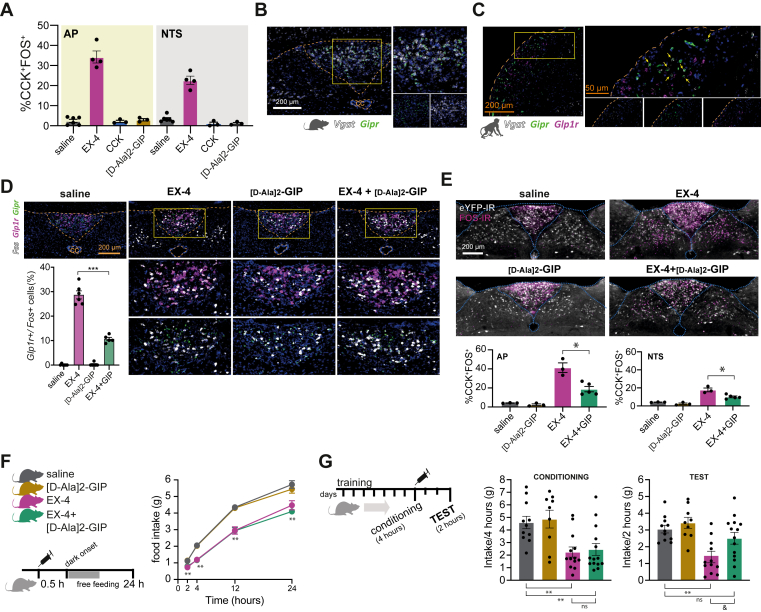
**GIPR agonism reduces the recruitment of *Glp1r*/CCK**^**AP/NTS**^**neurons and the conditioned taste avoidance elicited by Exendin-4.** (**A**) Quantification of CCK^AP/NTS^ neurons expressing FOS following an injection of EX-4, CCK, or [D-Ala2]-GIP (10, 10, and 100 μg kg^−1^, IP). (**B**) FISH labeling of endogenous *Gipr* and *Vgat* mRNA in the AP of the mouse. (**C**) FISH labeling of endogenous *Glp1r, Gipr,* and *Vgat* in the AP of the *Cynomolgus* monkey. (**D**) Representative FISH labeling and quantification of endogenous *Gipr*, *Glp1r,* and *Fos* mRNAs in the mouse AP following EX-4 (30 kg^−1^, IP) and [D-Ala2]-GIP (100 μg kg^−1^, IP) alone or in combination (n = 5–6; F_(3, 19)_ = 222.9, p < 0.0001, one-way ANOVA. Post hoc ∗∗∗p < 0.0001). (**E**) Quantification of CCK^AP/NTS^ neurons expressing FOS following EX-4 and [D-Ala2]-GIP alone, or in combination (n = 3–5; AP: F_(3, 10)_ = 30.73, p < 0.0001, one-way ANOVA; NTS: F_(3, 10)_ = 29.17, p < 0.0001. Post hoc ∗p < 0.001).(**F**) Effect of EX-4 and [D-Ala2]-GIP alone or in combination on food intake (n = 8–9; Treatment: F_(3, 31)_ = 21.91, p < 0.0001; Time: F_(1.853, 57.43)_ = 1214, p < 0.0001; Interaction: F_(9, 93)_ = 7.445, p < 0.0001, two-way ANOVA. Post hoc ∗∗p < 0.005) and (**G**) CTA (n = 9–14; Conditioning: F_(3, 43)_ = 7.176, p = 0.0005, one-way ANOVA. Post hoc ∗∗p < 0.005. Test: F_(3, 43)_ = 6.026, p = 0.0016, one-way ANOVA. Post hoc ∗∗p = 0.0021, ^&^p = 0.0321). Data are presented as mean ± SEM. See also, Supplemental Fig. 3.

Our data, so far, indicate that CCK^AP/NTS^ neurons represent an important functional exponent of the anorectic and nauseogenic effects of GLP-1RAs. Next, we asked whether CCK^AP/NTS^ neurons are responsive to other gastro-intestinal peptides — serving as a common cellular substrate for the integration of gastro-intestinal signals when these peptides are released postprandially. We found minimal activation of CCK^AP/NTS^ neurons when recombinant CCK (10 μg kg^−1^, IP) is administered systemically in *Cck*^Cre^::*eYFP* mice (Figure 4A). Next, we asked whether CCK^AP/NTS^ neurons are responsive to GIP administration, considering that GLP-1 and GIP are co-released and that dual GIP and GLP-1 receptor agonist peptides have been reported to have synergistic effects on body weight [38,39]. Since native GIP has a short half-life, we used a more stable GIP analogue, [D-Ala2]-GIP (100 μg kg^−1^, IP). We found that [D-Ala2]-GIP induces robust FOS-IR selectively in the AP (Figure 4D). However, almost none of these GIP-responsive cells are CCK^AP^ neurons (Figure 4A). These findings point to some specificity of CCK^AP/NTS^ neurons in integrating GLP-1 signaling and query the nature of the GIP-responsive neurons in the AP.

In accordance with the FOS study, using FISH, we found that *Gipr* mRNA is almost exclusively expressed in the AP and that it co-localizes with *Slc32a1* (the gene encoding for vGAT), a marker for GABAergic neurons (Figure 4B). Thus, while *Glp1r* mRNA is expressed predominantly in glutamatergic neurons in the AP and NTS — which include CCK^AP/NTS^ neurons — *Gipr* mRNA is expressed exclusively in GABAergic AP neurons. These observations are in line with a recently published single-cell transcriptomic analysis of AP neurons, which reported expression of *Gipr* mRNA in a subset of AP GABAergic cells [40]. Adding support to the translational significance of these findings, we confirm that *Gipr* mRNA, but not *Glp1r*, is expressed exclusively in GABAergic AP neurons also in the *Cynomolgus* monkey (Figure 4C).

Given that *Gipr* and *Glp1r* mRNAs are expressed in separate neuronal populations with inhibitory and excitatory phenotypes, respectively, we asked what the activation profile of GLP-1R-expressing neurons would be when EX-4 and [D-Ala2]-GIP are administered in combination. Using FISH, we found that EX-4 and [D-Ala2]-GIP, when administered alone, elicit distinct *Fos* mRNA expression in the AP and/or adjacent NTS, reflecting their respective receptor localizations (Figure 4D). Importantly, EX-4- and [D-Ala2]-GIP-induced *Fos* mRNA is receptor-dependent and is blunted in mutant mice lacking GLP-1R and GIPR, respectively (Supplemental Figs. 3A and B). However, when EX-4 and [D-Ala2]-GIP were co-administered, we observed that the fraction of activated *Glp1r* mRNA-expressing neurons in the AP was significantly reduced when compared to EX-4 treatment alone (Figure 4D), while the fraction of activated *Gipr* mRNA-expressing neurons is not different from that of [D-Ala2]-GIP treatment alone (Supplemental Fig. 3C). To confirm these data, we performed additional FOS studies and found that the fraction of CCK^AP/NTS^ neurons activated in response to EX-4 administration is reduced in mice receiving EX-4 and [D-Ala2]-GIP in combination (Figure 4E).

To understand the behavioral implications of this reduced recruitment of *Glp1r* mRNA-expressing neurons, we first measured feeding. [D-Ala2]-GIP has no effect on food intake, and it does not diminish the acute anorectic effect of EX-4 (Figure 4F).

Considering the clinical literature that highlights problems with treatment compliance due to the nausea-inducing effects of GLP-1R agonists, the observation that GIPR exists in a neuronal population that may functionally oppose neurons expressing the GLP-1R suggests an interesting dichotomy of function. Data from a Phase-2 clinical study of the dual GIP and GLP-1 receptor agonist tirzepatide in patients with Type-2 diabetes reveals a reduced incidence of nausea and vomiting compared to a GLP-1 agonist at the same clinical efficacy [39]. Thus, we asked whether GIPR agonism could oppose the aversive effects of GLP-1RAs in the CTA paradigm (Figure 4G). While EX-4 elicited CTA when administered alone, by contrast, we found that when EX-4 and [D-Ala2]-GIP are co-administered, the EX-4-induced CTA is significantly reduced (Figure 4G). These findings suggest that the negative valence associated with GLP-1RAs is scalable and, to some extent, dissociable from the anorectic effect. These findings are also in line and expand on recent findings showing that GIPR activation reduces emesis induced by GLP-1RA in musk shrews [41]. Considering that the tolerability of GLP-1RAs is reduced because of drug-related nausea, the findings that GIP receptor activation opposes the aversive effect of GLP-1RAs could have significant therapeutic implications.

### 3.5 Limitation of study

We were unable to directly assess whether there were distinct contributions of CCK^AP^ versus CCK^NTS^ neurons to the anorectic and nausea-promoting effects of GLP-1RAs. CCK^AP/NTS^ neurons form an anatomical continuum between the AP and the NTS, making it difficult to target the two neuronal subsets separately using surgical approaches. However, it is also possible that both neuronal subsets contribute to mediating the effects of GLP-1RAs. In general, in addition to sharing neurochemical identities, AP and NTS neurons also have a common target region (i.e., the PBN) and can be similarly activated by both circulating and electrical signals (e.g., gut hormones, vagal inputs), suggesting that CCK^AP^ and CCK^NTS^ neurons could also be seen as part of a wider dorsal vagal complex neuronal population. The identification of additional discrete molecular markers and alternative targeting approaches, such as new intersectional genetic approaches, will help address this issue in future studies.

## Author contribution

GD’A, SML, MA and PE conceived the project, supervised the experiments and their analyses. GD’A wrote the manuscript with inputs from MA, PE, TC, and SML. MA, AC, NN, IC, JH, BS, MBB, LVT,GA, and GD’A, performed experiments and data analysis. DJH provided reagents and materials. All authors contributed to the editing of the final manuscript.

## Grant support

This work was funded by an MRC Career Development Award (MR/P009824/1 and MR/P009824/2) to GD’A, as well as an MRC grant to SML/GD’A (MR/T032669/1), a 10.13039/501100000268BBSRC grant to SML (BB/M001067/1), and an additional direct contribution from Eli Lilly. D.J.H. was supported by 10.13039/501100000265MRC (MR/N00275X/1 and MR/S025618/1), 10.13039/501100000361Diabetes UK (17/0005681), and the European Research Council (ERC) under the European Union's Horizon 2020 research and innovation programme (Starting Grant 715884 to D.J.H.). AC was supported for part of this project by a travel grant from the 10.13039/501100015272Italian Society of Pharmacology and a fellowship from the Veronesi Foundation (Italy).
