# Supplementary material for: Anorectic and aversive effects of GLP-1 receptor agonism are mediated by brainstem cholecystokinin neurons, and modulated by GIP receptor activation
Source: Mol Metab. 2021 Nov 26;55:101407. doi: 10.1016/j.molmet.2021.101407 (PMC8689241; doi:10.1016/j.molmet.2021.101407)
Supplement: Supplementary file 1 [file mmc1.pdf]

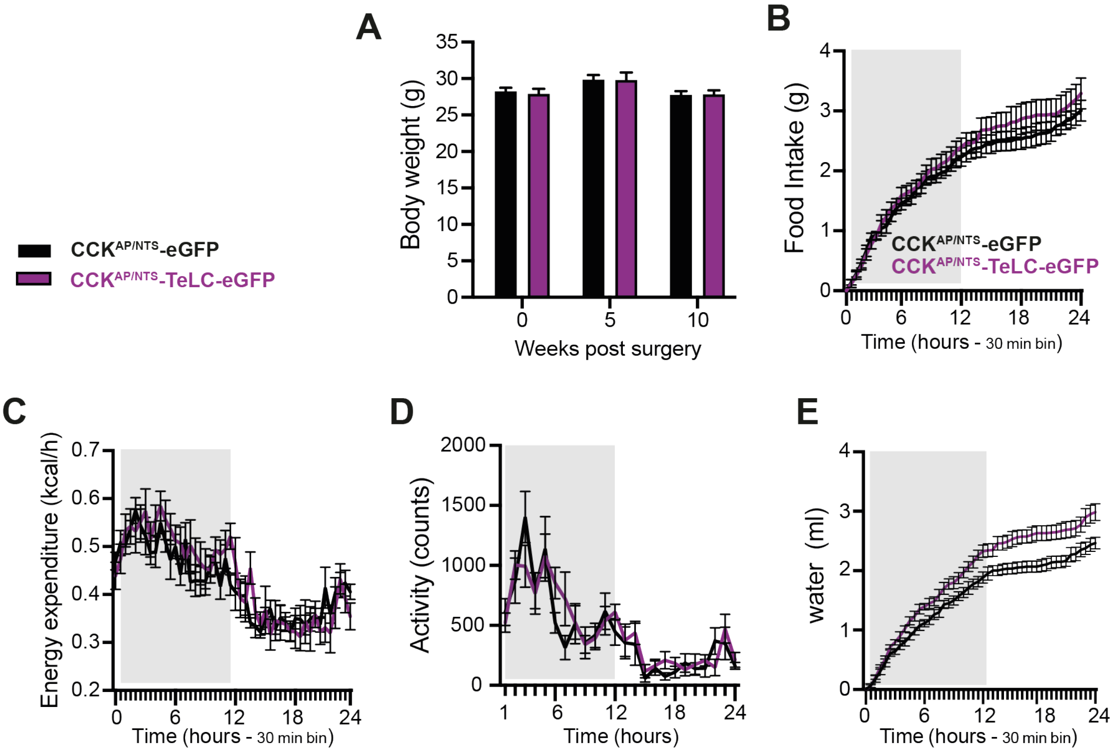

Supplemental Figure 1. (**A**) Body weight in CCK$^{AP/NTS}$-eGFP and CCK$^{AP/NTS}$-TeLC-eGFP mice before and following a targeted injection of AAV-eGFP or AAV-TeLC-eGFP in the AP/NTS via stereotaxic. (**B-E**) Representative recordings over 24 hours of (**C**) food intake, (**D**) energy expenditure, (**E**) locomotor activity, and (**F**) water intake ($F_{(1,14)}$= 11.80, p= 0.0040) in CCK$^{AP/NTS}$-eGFP and CCK$^{AP/NTS}$-TeLC-eGFP mice. Data are presented as mean ± SEM. See also Main Figure 2.

Supplemental Figure 2

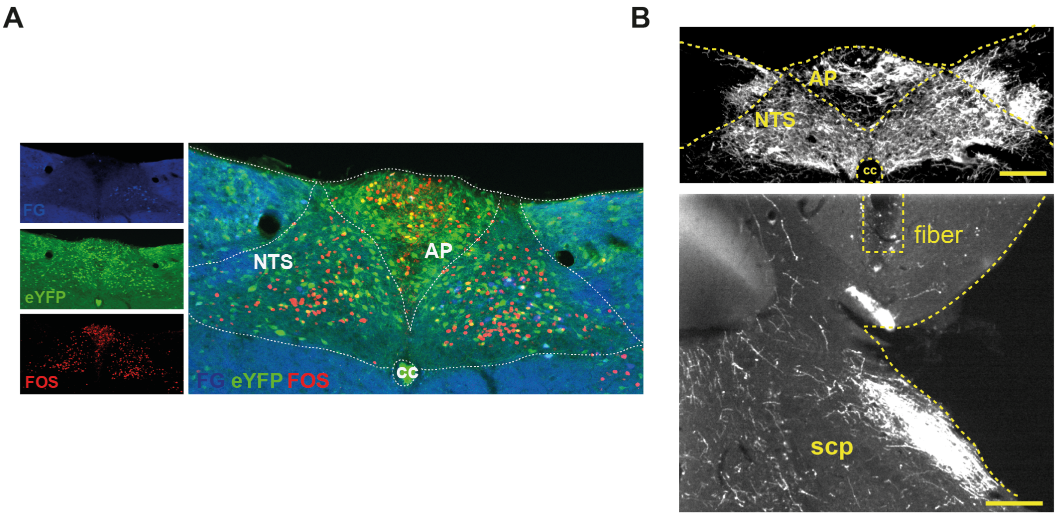

Supplemental Figure 2. (**A**) Representative image of $CCK^{AP/NTS}$ neurons projecting (→) to the paraventricular nucleus of the hypothalamus (PVH) identified using Fluoro-Gold (FG) retrograde tracing. (**B**) Top panel: representative images of $CCK^{AP/NTS}$ neurons expressing ChR2-eYFP. Bottom panel: eYFP+ $CCK^{AP/NTS}$ neuronal projections in the Parabrachial nucleus (PBN) and location of the optic fiber placement. AP, Area Postrema; NTS, nucleus of the solitary tract; scp, superior cerebellar peduncle. See Main Figure 3.

Supplemental Figure 3

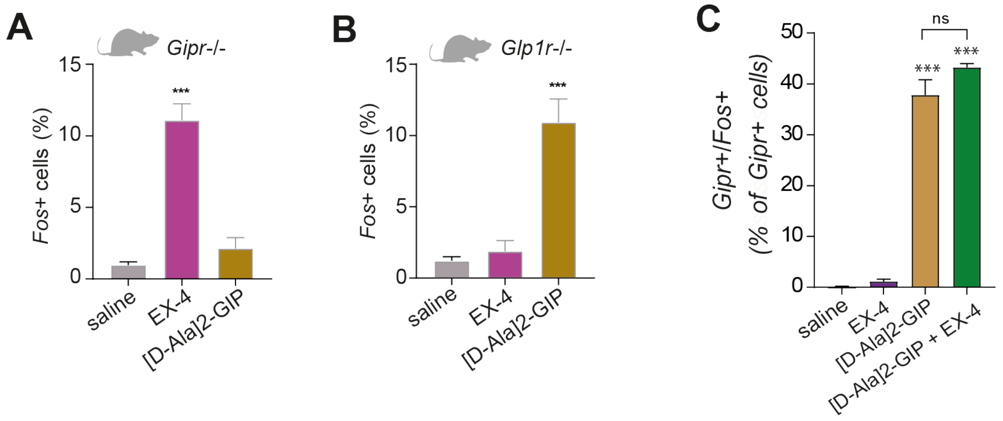

Supplemental Figure 3. (**A**) Quantification of *Fos* mRNA expressing cells in the AP following EX-4 (30 kg^{-1}, IP) and [D-Ala2]-GIP (100µg kg^{-1}, IP) in knock-out mice lacking the *Gipr*. (**B**) Quantification of *Fos* mRNA expressing cells in the AP following EX-4 (30 kg^{-1}, IP) and [D-Ala2]-GIP (100µg kg^{-1}, IP) in knock-out mice lacking the *Glp1r*. (**C**) Quantification of *Glp1r* mRNA expressing cells co-expressing *Fos* mRNA in the mouse AP following EX-4 (30 kg^{-1}, IP) and [D-Ala2]-GIP (100µg kg^{-1}, IP) alone or in combination. One way ANOVA followed by Tukey's group comparison was performed for all panels, ***p<0.001 compared to saline group, ns, not significant. Data are presented as mean ± SEM. See also Main Figure 4.
